# The Nineteenth and Twentieth Centuries

**Published:** 1918-08-10

**Authors:** 


### The London Hospital Nurses.

### THE NINETEENTH AND TWENTIETH CENTURIES.

Last week we published a letter from Miss Helena McKinley, a trained nurse and matron of some years' standing, and on the same page letters from Major Chapple, M.P., and Viscount Knutsford under the above heading. Miss McKinley was very severe upon certain matters which she recited as affecting the nurses at the London Hospital. The most material one was as follows:—" But is it true that they [the probationer nurses] get even two years' work in the wards? I know many 'Londoners' who have complained of lack of work and teaching, and who have been sent out private nursing under twelve months." Major Chapple quoted the following words, taken from the evidence of Lord Knutsford before a Select Committee of the House of Commons on the Registration of Nurses, 1904:—" Nurses are not sent out now until they have done two years in the hospital except in exceptional circumstances." Lord Knutsford in his letter referring to this evidence states it is what he " said fourteen years ago of what had happened some years before even that remote date [1904], and happened under very, very exceptional circumstances, fully explained at the time. He [Major Chapple] knows all this as I told it to him before when he made the same untrue statement." Now Lord Knutsford's letter therefore officially disposes, as chairman of the London Hospital, of Miss Helena McKinley's complaint—that London Hospital nurses suffer from " lack of work and teaching, and have been sent out private nursing under twelve months." We have been in the closest touch with the London Hospital authorities, who have invariably given us the fullest information on every point, as occasion has arisen, during many years. Miss Helena McKinley urges that it has been common cause of comment amongst nurses that we have practically always supported Lord Knutsford, and it has consequently been most difficult for them to understand this attitude on our part. The facts we have just set forth from published letters in The Hospital supply a good reason for the attitude in question.

It has been our privilege for nearly fifty years, commencing from a time long before the day of trained nurses, to have been associated with hospitals and nurses, and to have devoted much time, thought, and energy to everything calculated to improve the position and raise the standard of both. Miss McKinley has appealed to us to " take up the cause of the probationer nurse and to secure for her what she is too young and inexperienced to know for herself when she enters for her training." " Ierne " has been eloquent of late on the silence of nurses. " Why are nurses dumb? " " Ierne " asks, and at " Ierne's " instigation many have become articulate. It is a remarkable fact that during the whole discussion which has taken place on the system of training nurses at the London Hospital we can recall no complaint that has been addressed to us by a probationer nurse.

Lord Knutsford, in his reply to Major Chapple just referred to, states distinctly that " over one hundred of the nurses on the London private staff could leave to-morrow if the conditions of their service were unfair." Lord Knutsford has testified that the statement affecting probationers does not apply to the conditions of training in the London Hospital to-day. There is no evidence adduced directly by the statements of any probationer nurse trained at the London Hospital to the contrary. Our columns have been open to all matrons, sisters, and nurses who are in want of information or help, or who have any just complaint to make of the conditions of their training, or of the conditions under which they are employed. In these circumstances, failing any evidence from present-day probationers to the contrary, and having before them an official statement of Lord Knutsford, chairman of the London Hospital, published on page 397 of The Hospital of the 3rd inst., it is due to the nursing profession, in the absence of any such complaints, that the London Hospital should no longer suffer amongst trained nurses from floating statements, which have never been demonstrably proved to relate to its conditions of nurse training at the present time.

In putting Miss McKinley's complaint and Lord Knutsford's reply on the same page we have endeavoured to act with strict fairness and impartiality towards both sides. We live in the Twentieth Century, not the Nineteenth—a fact which some complainants appear to have forgotten.

